# Activation of human CD8+ T-cells with nitroso dapsone-modified HLA-B*13:01-binding peptides^1^

**DOI:** 10.4049/jimmunol.2200531

**Published:** 2023-04-15

**Authors:** Mubarak Almutairi, Adam Lister, Qing Zhao, James Line, Kareena Adair, Arun Tailor, James Waddington, Elsie Clarke, Joshua Gardner, Paul Thomson, Nicolas Harper, Yonghu Sun, Lele Sun, David A. Ostrov, Hong Liu, David J. MacEwan, Munir Pirmohamed, Xiaoli Meng, Furen Zhang, Dean J Naisbitt

**Affiliations:** † MRC Centre for Drug Safety Science, Dept. Molecular & Clinical Pharmacology, University of Liverpool, Liverpool, UK; ‡ Shandong Provincial Hospital for Skin Diseases & Shandong Provincial Institute of Dermatology and Venereology, Shandong First Medical University & Shandong Academy of Medical Sciences, Jinan, Shandong, China; § Department of Pathology, Immunology and Laboratory Medicine, University of Florida, College of Medicine, Gainesville, Florida, USA

## Abstract

Previous studies have shown that cysteine-reactive drug metabolites bind covalently with protein to activate patient T-cells. However, the nature of the antigenic determinants that interact with HLA, and whether T-cell stimulatory peptides contain the bound drug metabolite has not been defined. Since susceptibility to dapsone hypersensitivity is associated with the expression of HLA-B*13:01, we have designed and synthesized nitroso dapsone-modified, HLA-B*13:01 binding peptides and explored their immunogenicity using T-cells from hypersensitive human patients. Cysteine-containing 9mer peptides with high binding affinity to HLA-B*13:01 were designed (AQDCEAAAL [Pep1], AQDACEAAL[Pep2] and AQDAEACAL [Pep3]) and the cysteine residue was modified with nitroso dapsone. CD8+ T-cell clones were generated and characterized in terms of phenotype, function and cross-reactivity. Autologous antigen presenting cells and C1R cells expressing HLA-B*13:01 were used to determine HLA restriction. HPLC/LCMS confirmed that nitroso dapsone-peptides were modified at the appropriate site and were free of soluble dapsone and nitroso dapsone. Antigen presenting cell HLA-B*13:01-restricted nitroso dapsone-modified Pep1 (n=124) and Pep3 (n=48) responsive CD8+ clones were generated. Clones proliferated and secreted effector molecules with graded concentrations of nitroso dapsone-modified-Pep1 or Pep3. They also displayed reactivity against soluble nitroso dapsone, which forms adducts *in situ*, but not with the unmodified peptide or dapsone. Cross-reactivity was observed between nitroso dapsone-modified peptides with cysteine residues in different positions in the peptide sequence. These data characterise a drug metabolite hapten CD8+ T-cell response in an HLA risk allele-restricted form of drug hypersensitivity and provide a framework for structural analysis of hapten HLA binding interactions.

## Introduction

Dapsone is a widely used antibacterial agent and is frequently prescribed for the treatment of leprosy. However, exposure to dapsone is associated with the development of a hypersensitivity syndrome, characterised by fever, skin rash, hepatitis, and generalized lymphadenopathy, in 0.5-3.6% of treated patients (1, 2). Recent studies have shown that expression of human leukocyte antigen (HLA)^2^-B*13:01 is associated with increased risk of dapsone hypersensitivity (3–6).

Dapsone contains aromatic amine groups that are susceptible to acetylation by *N*-acetyltransferase enzymes, a process of detoxification that limits the lifespan of the drug. The aromatic amine groups also undergo cytochrome P450-mediated hydroxylation yielding dapsone hydroxylamine (7). Nitroso dapsone is generated via auto-oxidation of the hydroxylamine metabolite. Nitroso dapsone is protein-reactive and covalently modifies cysteine residues on proteins (8–11).

Peripheral blood mononuclear cells (PBMC) from hypersensitive patients expressing HLA-B*13:01 are stimulated to proliferate with both dapsone and its nitroso metabolites (12, 13). Through the generation of clones, HLA class II-restricted CD4+ and HLA-class I- and II-restricted CD8+ T-cells were shown to be stimulated with dapsone and nitroso dapsone via different pathways (13, 14). The T-cell response to parent drug and metabolite was polyclonal with T-cells displaying a variety of TCR sequences (14). Dapsone interacts directly with HLA to trigger T-cell receptors, whereas nitroso dapsone triggers T-cell receptors though a pathway dependent on antigen processing, presumably through the formation of protein adducts. Both dapsone and nitroso dapsone interact with multiple HLA class I proteins to activate CD8+ T-cells, which makes it challenging to focus research on the drug HLA-B*13:01 interaction and specifically determine whether drug metabolite-modified peptides activate T-cells in an HLA allele-restricted manner. Therefore, the aim of this work was to (1) design HLA-B*13:01 binding peptides that contain a reactive cysteine residue (2), generate nitroso dapsone-modified peptides that are free of dapsone and the nitroso metabolite, and (3) explore immunogenicity of the peptides using autologous antigen presenting cells (APC) and APC transfected with the single HLA-B allele HLA-B*13:01.

## Methods

### Synthesis of drug modified peptides

**Figure 1:**
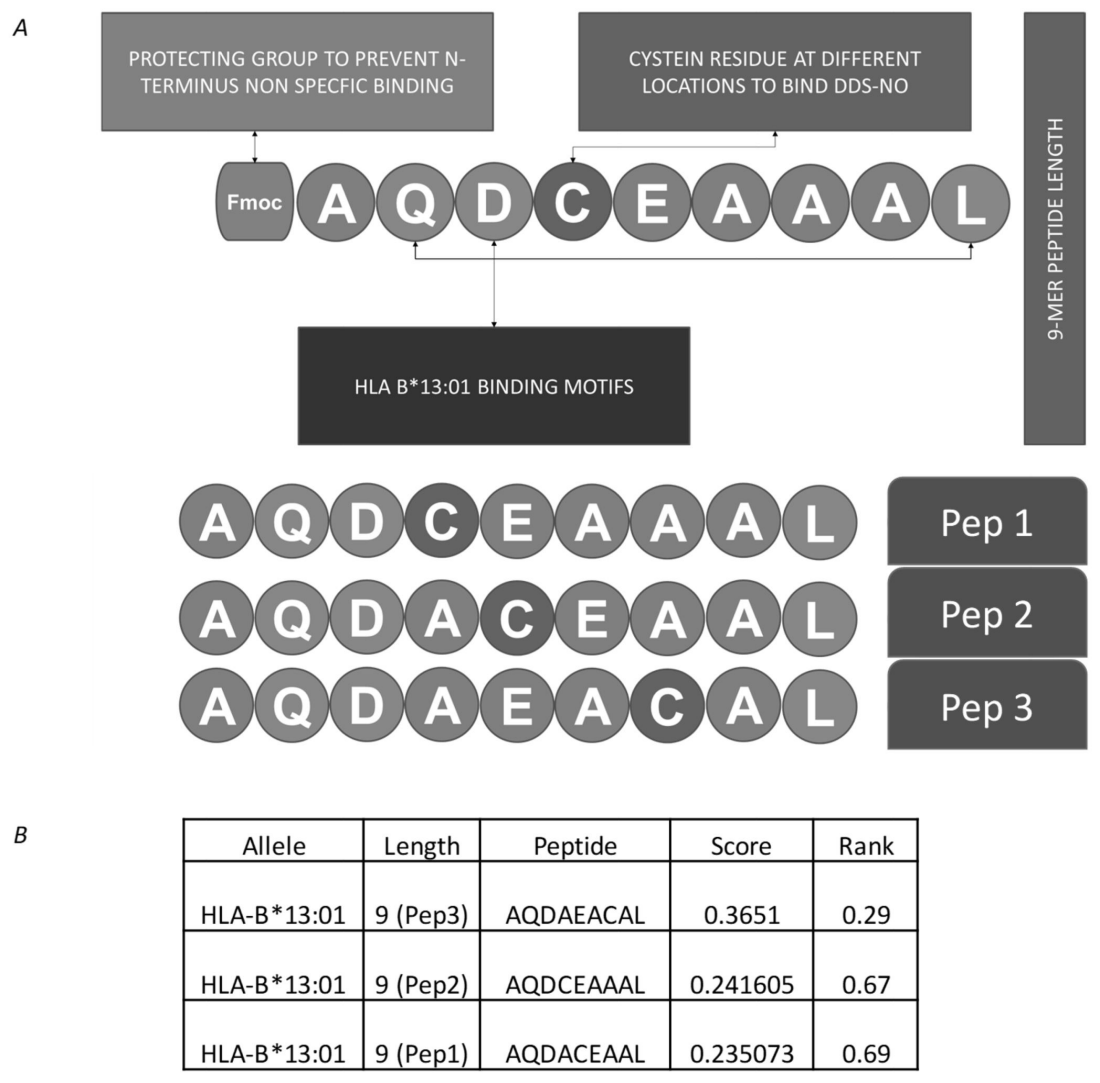
Strategy to design nitroso dapsone-modified HLA B*13:01 peptides. (A) Schematic illustrating the strategy used to design and synthesize nitroso dapsone-modified HLA binding peptides; (B) all three peptides showed favourable binding to HLA-B*13:01 (a percentile rank of < 1 as predicted by NetMHC, Immune Epitope Database).

Three 9mer peptides were identified as potential HLA-B*13:01 high affinity binders using the MHC binding prediction tool obtainable at www.iedb.org (15). Peptides were designed to each contain a single cysteine residue at sites distal from the HLA-B*13:01 binding motifs (glutamine at P2 and leucine at P9), as previous studies have shown that nitroso dapsone binds covalently to cysteine (11). Poly-alanine was chosen as peptide backbone to minimize the interaction between peptides and TCR. Cysteine was inserted within the binding motif to generate positional derivatives. To improve peptide solubility, glutamic acid and aspartic acid were also added. Aspartic acid is also a secondary anchor in position 3. All three peptides showed favourable binding to HLA-B*13:01 (a percentile rank of < 1 as predicted by NetMHC, Immune Epitope Database). The strategy for peptide design and predicted HLA-B*13:01 binding affinity is summarized in Figure 1A and B. Peptides were synthesized with an Fmoc protecting group at the N-terminus to avoid a non-specific reaction between nitroso dapsone and the primary amine of the peptide. The protecting group was removed before any functional analyses. Characterisation of the peptides was performed by mass spectrometry (16).

Fmoc-peptide 1 (Pep1, Fmoc-AQD**C**EAAAL), -peptide 2 (Pep2, Fmoc-AQDA**C**EAAL), and peptide 3 (Pep3, Fmoc-AQDAEA**C**AL) were purchased from SynPeptide Co Ltd (SynPeptide CO., LTD, Shanghai, China). Purity was initially confirmed by HPLC and identity was confirmed by mass spectrometry. Peptides were incubated with nitroso dapsone at a 4:1 molar ratio in 70% ACN/30% H_2_O for 16-24 hrs at 37°C. Nitroso dapsone-modified Fmoc-peptides were then analysed by HPLC with a Gemini^®^ 5 μm NX-C18 110 Å, LC Column 250 x 4.6 mm (Phenomenex, Macclesfield, Cheshire, United Kingdom) connected to an Agilent 1260 Infinity Quaternary LC (Agilent Technologies, Stockport, Cheshire, United Kingdom). The fractions containing modified peptides were then analysed by mass spectrometry using a TripleTOF 6600 (AB Sciex) mass spectrometer. Fmoc was removed by incubating of conjugated peptides with 20% piperidine in DMF at room temperature for 3 h. The nitroso dapsone-modified peptides were analysed again by HPLC to confirm removal of the Fmoc protecting group. Fractions were collected and analysed by mass spectrometry.

#### Mass spectrometric characterisation of dapsone nitroso modified peptides

Nitroso dapsone-modified peptides were characterized using a TripleTOF 6600 (AB Sciex) mass spectrometer. Briefly, samples were delivered into the mass spectrometer by automated in-line reversed phase liquid chromatography, using an Eksigent NanoLC 400 System (AB Sciex) mounted with a trap and analytical column (15 cm X 75 μm). A NanoSpray III source was fitted with a 10 μm inner diameter PicoTip emitter (New Objective). A gradient of 2–50% (v/v) acetonitrile/0.1% (v/v) formic acid over 90 min was applied to the column at a flow rate of 300 nl/min. Spectra were acquired automatically in positive ion mode using information dependent acquisition, using mass ranges of 400–1600 Da in MS and 100–1400 Da in MS/MS. Up to 25 MS/MS spectra were acquired per cycle (approximately 10 Hz) using a threshold of 100 counts per s, with dynamic exclusion for 12 s and rolling collision energy.

#### Quantification of drug molecules remaining in the peptide fractions

Free dapsone/dapsone nitroso remaining in the drug-modified peptide fractions were quantified by mass spectrometry. Calibration standards were prepared at the following concentrations (5-500 nM). All samples were diluted in 0.1% formic acid prior to analysis and spiked with the internal standard sulfamethoxazole (250 nM). Samples and standards were analysed immediately by a QTRAP5500 mass spectrometer (AB Sciex) coupled with an Ultimate 3000 LC system (Dionex Corporation, Sunnyvale, California). The multiple reaction monitoring transitions for each analyte were as following: dapsone 249.1/156.1, dapsone nitro 263.1/156.1, and the internal standard 254.1/156.1. Other mass spectrometry (MS) parameters, such as voltage potential and collision energy were optimized to achieve great sensitivity. Data acquisition and quantification were performed using Analyst 1.5 software.

### HLA-B*13:01 modelling

A homology model of HLA-B*13:01 was built from HLA-B*52:01 (96% sequence similarity, PDB: 3w39) using SWISS-MODEL. GOLD 5.2 (CCDC software) was used to dock the peptides AQDCEAAAL, AQDWEAAAL, and AQDC(DDS)EAAAL within the binding groove, with the binding site defined as 10 Å around the binding point. The binding point was further refined with key amino acid residues within B pocket (Tyr7, Tyr9, Phe22, Thr24, VAL34, Met45, Glu63, Ile66, Ser67, Thr69 and Asn70) and F pocket (Tyr74, Asn77, Thr80, Tyr84, Ile95, Arg97, Asp114, Leu116, Tyr123, Ile124, Thr143, Lys146, Trp147). A generic algorithm with ChemPLP as the fitness function was used to generate 10 binding modes per ligand. Default settings were retained for the “ligand flexibility”, “fitness and search options”, and “GA settings.

### Cell Culture Medium

PBMC were cultured in R9 medium (RPMI-1640 medium supplemented with 100 U/ml streptomycin, 100 mg/ml penicillin, 25 mg/ml transferrin, 2 mM L-glutamine 10% human AB serum, and 25 mM HEPES buffer). Medium was supplemented with IL-2 to maintain T-cell lines and clones. RPMI-1640 supplemented with fetal bovine serum, penicillin (100 U/ml), streptomycin (0.1 mg/ml), 25 mM HEPES buffer and 2 mM L-glutamine was used for EBV-transformed B-cell cultures.

### Human subjects

Venous blood samples (50ml) were taken from 2 dapsone hypersensitive patients with positive (i) dapsone patch test and (ii) dapsone and nitroso dapsone lymphocyte transformation test as reported previously (14). Patient 8 (female, 28 years old at time of adverse event) displayed fever and abnormal liver function tests following 17-day exposure to dapsone. Patient 14 (male, 24 years old at time of adverse event) displayed fever, skin rash (erythema) and abnormal liver function tests following 21-day exposure to dapsone. Both individuals expressed HLA-B*13:01; the full patient HLA profiles are available in Zhao et al (14). Approval for the study was acquired from Shandong Provincial Institute of Dermatology and Venereology and informed written consent was obtained. A material transfer agreement was signed prior to transport of PBMC to Liverpool.

#### Anti-dapsone antibody production

Ovalbumin-dapsone conjugates were prepared by the reaction of nitroso dapsone with ovalbumin at a molar ratio of 10:1 (drug to protein) using methods as previously described (17). Antibody production was performed by Kaneka Eurogentec S. A. (Belgium) using a speedy 28-polyclonal package. Detailed information is available online (eurogentec.com).

### Generation of nitroso-dapsone-modified peptide-responsive T-cell clones

PBMC (1 x 10^6^/well) were cultured in a 48-well plate with nitroso dapsone-modified Pep1 (patient 8 and 14) and Pep3 (patient 14 only due to limitations on the availability of the modified peptide) for 14 days to enrich the number of responsive T-cells prior to serial dilution. Peptide concentrations (10-50 μM) were selected based on a lack of intrinsic toxicity and no inhibition of phytohaemagglutinin (PHA)-treated healthy donor PBMC proliferation. Pep2 was reserved for crossreactivity studies as it was synthesized in low yield. T-cell clones were generated by serial dilution and repetitive mitogen-driven expansion. Briefly, irradiated allogenic PBMC (5 × 10^4^ cells/well) in medium containing PHA and IL-2 were added to 96 well U bottom plates. T-cells were then diluted and added to the PBMC mixture at 0.3, 1 and 3 cell/well. Cultures were incubated for 14 days (37°C / 5% CO_2_) and medium was supplemented with IL-2 every 2 days. On day 14, the growing clones were restimulated with PHA and irradiated allogenic PBMC (5 × 10^4^ cells/well) in IL-2 containing medium and expanded for a further 14 days prior to testing for peptide specificity.

### Antigen presenting cells

PBMC from the hypersensitive patients were cultured with supernatant from EBV-producing B-958 cells in the presence cyclosporine-A to generate immortalized autologous B-cell lines using established methods (14).

To generate a HMy2.C1R-HLA-B*13:01-P2A-B2M cell line, the pLJM1-EGFP plasmid (gift from David Sabatini Addgene plasmid # 19319) was modified by replacing the EGFP with a Multiple Cloning Site, adding an Eμ enhancer (pLJM1-Eμ-SFFV-NewMCS) and a P2A sequence. B2M was PCR amplified from cDNA from a volunteer and ligated into the distal end of the P2A sequence using restriction sites Age1/SnaB1. HLA-B*13:01 was PCR amplified from the HLA-B*13:01 pcDNA3.1(+) plasmid and ligated into the proximal end of the P2A sequence using restriction sites PME1 and EcoR1 to create the pJLM1-HLA-B*13:01-P2A-B2M plasmid. The pJLM1-HLA-B*13:01-P2A-B2M plasmid was transfected into competent E. coli and colonies grown on agarose plates under carbenicillin selection overnight at 37°C. Positive colonies were identified by colony PCR and grown up overnight in a shaking incubator at 37°C. Plasmids were purified using the QIAprep Spin Miniprep Kit (Qiagen). Correct integration was confirmed by Sagner sequencing. The HEK293 cell line was used as the machinery to generate 2nd generation lentivirus. HLA-B*13:01-P2A-B2M lentivirus was collected from the supernatant 96 h post transfection. HMy2.C1R cells were transduced with the HLA-B*13:01-P2A-B2M lentivirus supernatant and selected for positive transduction with puromycin. HMy2.C1R cells (ECACC 94050320), an EBV transformed B-cell line lacking HLA-B expression, was purchased from the UK Health Security Agency culture collections (https://www.culturecollections.org.uk).

### Specificity testing of T-cell clones

Expanded T-cell clones (5 × 10^4^ cells/well) were incubated with irradiated autologous antigen presenting cells (EBV-transformed B-cells; 1 × 10^4^ cells/well) and modified peptides (10 μM) for 48 h in duplicate cultures, and proliferation was measured through addition of [^3^H]-thymidine (0.5 μCi/well) for the final 16 h of the culture period. Peptide-free medium was used as negative control.

### Characterization of drug-modified peptide-responsive T cell clones

T cell clones were phenotyped using flow cytometry for the CD4+ (CD4-APC (clone RPA T4)) and CD8+ surface receptors (CD8-PE (clone HIT8a)). T-cell clones (5 × 10^4^ cells/well) were tested in dose-response studies for cross-reactivity with irradiated antigen presenting cells (1 × 10^4^ cells/well) and nitroso dapsone-modified peptides (Pep1, 2 and 3; 1 to 100 μM) in triplicate cultures. Unmodified peptides subjected to the same culture conditions and purification steps were used as a negative control (1 to 100 μM). Clones were also cultured with soluble nitroso dapsone (1-40 μM; higher concentrations induced toxicity). Proliferation of the clones was measured by addition of [^3^H]-thymidine for the final 16 hrs of the experiment.

The secretion of IFN-γ from the clones was assessed using ELIspot. T-cell clones were incubated with antigen presenting cells in the presence and absence of nitroso dapsone-modified peptides and other study compounds in IFN-γ antibody-coated ELIspot plates (37°C, 5% CO_2_) for 48 hrs. Plates were then developed according to the manufact rer’s instructions (Mabtech, Stockholm) and spots were counted using an AID ELIspot reader. To confirm levels of cytokine produced, the supernatant of 4 clones (2 drug-specific, 2 not drug specific) was analysed using a cytokine bead array according to the manufacturer’s instructions (LEGENDplex, Biolegend Custom Human 11-plex panel). Briefly, supernatant pooled from triplicate wells for each clone was added to specific antibody-coated beads forming an analyte-antibody complex. After washing, a biotinylated detection antibody cocktail was added which bound to the specific analyte-antibody complexes. Streptavidin-phycoerythrin was subsequently added which bound to the biotinylated detection antibodies, providing fluorescent signal intensities in proportion to the bound analyte amount. Fluorescent signals were measured using BD FACSCanto II and analysed using LEGENDplex data analysis software where concentrations of each analyte are determined using a standard curve generated in the same assay.

In order to determine whether the detected activation of clones with nitroso dapsone-modified peptides was due to residual dapsone or nitroso dapsone or degradation of the peptides and liberation of free dapsone, clones (5 × 10^4^ cells/well) were cultured with irradiated antigen presenting cells (1 × 10^4^ cells/well) (i) in the presence of soluble dapsone (125-500 μM) and proliferation was measured by addition of [^3^H]thymidine; and (ii) in the presence of nitroso dapsone-modified Pep1 or soluble nitroso dapsone and glutathione (1 mM), which binds covalently to the nitroso metabolite preventing protein binding (11), and proliferation was measured by addition of [^3^H]thymidine. The irreversible binding of nitroso dapsone to antigen presenting cells was measured in the presence and absence of glutathione (1 mM) using immunofluorescence staining with an anti-dapsone antibody.

To explore the importance of HLA proteins in T-cell activation, T-cell clones (5 × 10^4^ cells/well) were cultured with Pep1 or Pep3 (i) in the absence of antigen presenting cells; (ii) in the presence of C1R-B*13:01 or C1R-parental antigen presenting cells (1 × 10^4^ cells/well); and (iii) in the presence of C1R-B*13:01 antigen presenting cells pre-treated with either isotype (IgG1) or HLA class-I (DX17) or HLA class-II (Tu39) blocking antibodies for 30 min. T-cell proliferation or IFN-γ release were measured using [^3^H]thymidine or ELIspot, respectively.

## Results

### Synthesis and analysis of nitroso dapsone-modified peptides

**Figure 2:**
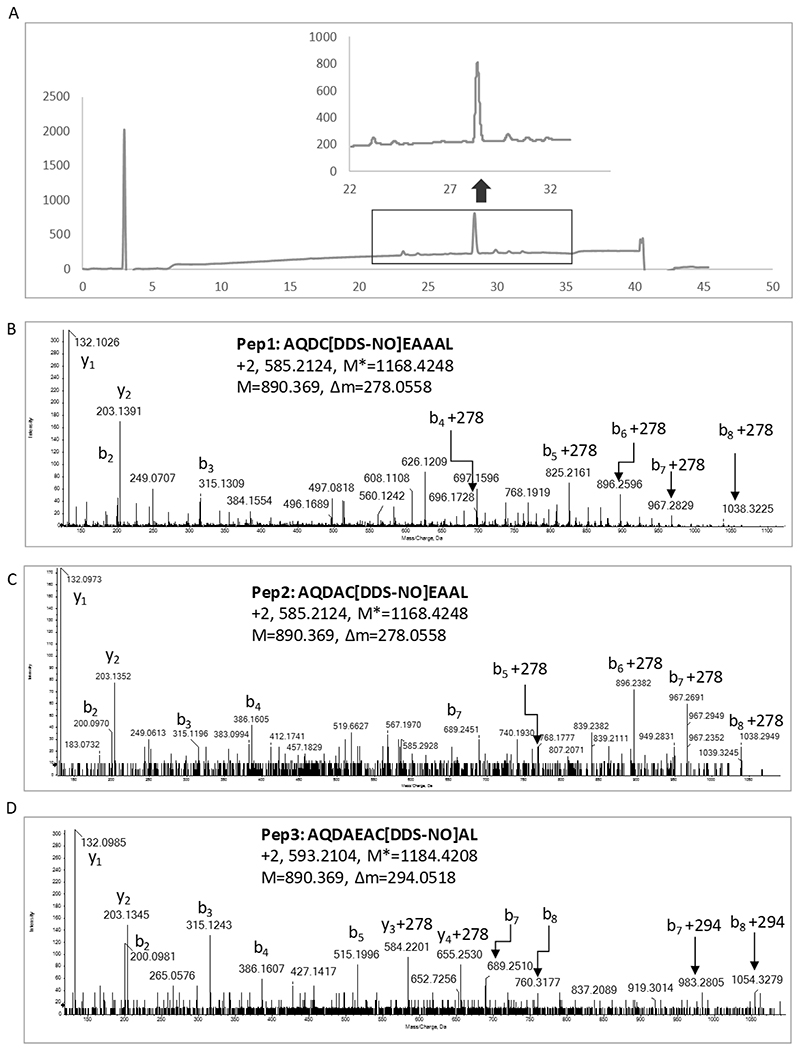
Characterization of nitroso dapsone modified HLA B*13:01 peptides. (A) HPLC analysis shows that the conjugated peptide (representative trace of Pep1 shown) is essentially free of dapsone, nitroso dapsone and unmodified peptide. (D-D) Mass spectrometric analysis shows characteristic peptide fragment ions with mass addition of 278 amu to confirm the site of nitroso dapsone modification on Pep 1 (B) and Pep2 (C). A *N*-hydroxyl sulphonamide adduct with a mass addition of 294 amu on Pep3 was also detected (D).

**Figure 3:**
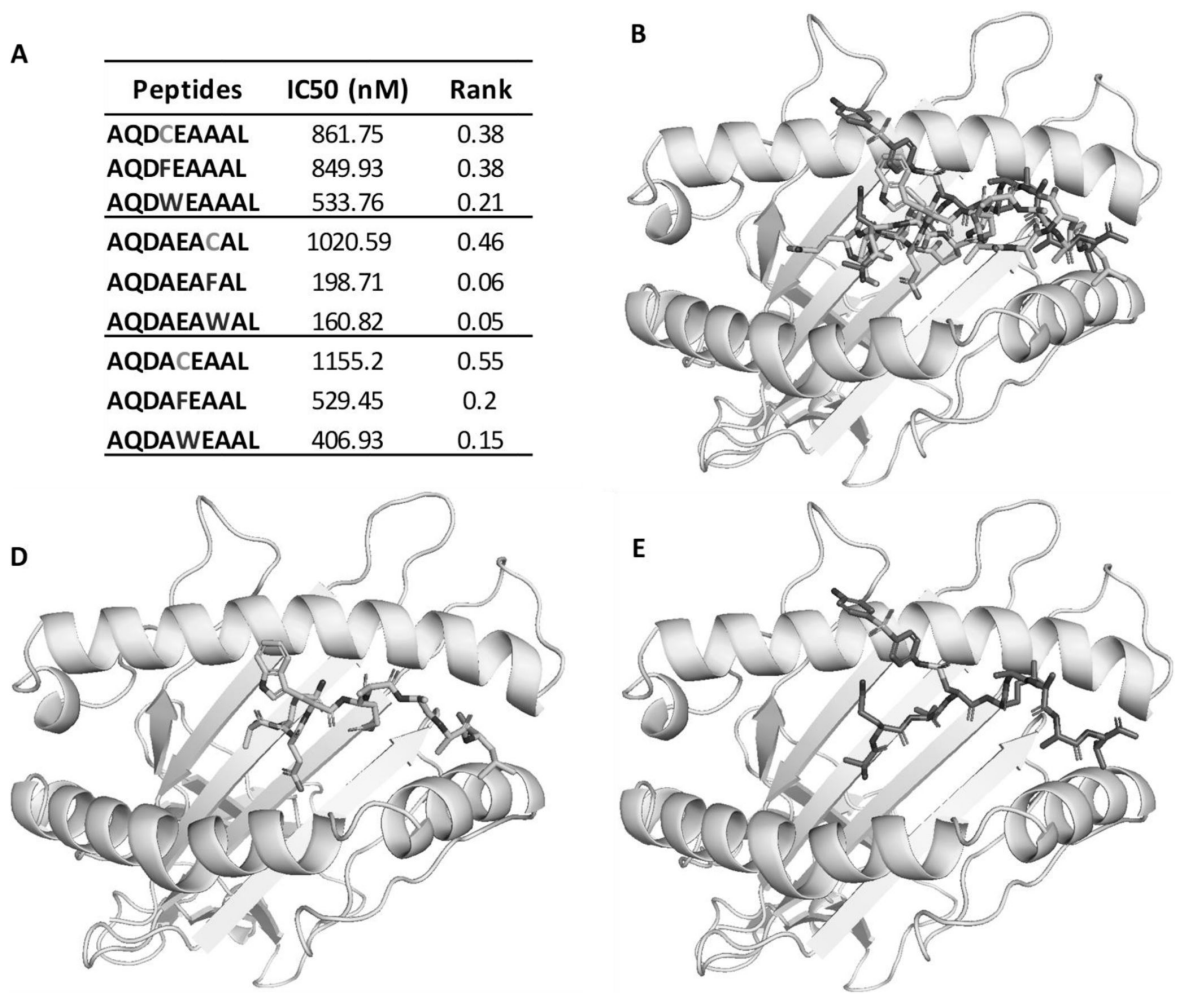
*In silico* prediction of the binding of dapsone nitroso modified peptides to HLA-B*13:01. (A) All three designer peptides containing a cysteine residue without dapsone nitroso modification showed favourable binding to HLA-B*13:01; replacement of the cysteine residue with bulky aromatic amino acids (F and W) to mimic dapsone nitroso modification increases the binding of peptides to HLA-B*13:01. The binding affinity to HLA-B*13:01 was predicted by NetMHCpan BA4.1, Immune Epitope Database. (B) The predicted conformation of AQDCEAAAL, AQDWEAAAL, and AQDC(DDS)EAAAL in complex with HLA-B*13:01, a homology model generated from HLA-B*52:01 (PDB: 3W39) by SWISS-MODEL. (C-D) Dapsone nitroso modified peptide AQDC(DDS)EAAAL (D) is predicted to bind to HLA-B*13:01 in the similar conformation as peptide AQDWEAAAL (C), with the bulky aromatic groups pointing out of the binding groove. All images are illustrated by PyMOL (The PyMOL Molecular Graphics System, Version 1.3 Schrödinger, LLC.).

HLA-B*13:01 designer peptides were created by incorporating HLA-B*13:01 anchor residues and a cysteine residue (Figure 1A). These peptides are predicted as strong binders using the MHC binding prediction tool (pep1 0.862 μM; pep2 1.155 μM; pep3 1.175 μM; Figure 1 B). Nitroso dapsone-modified peptides were prepared from conjugation of *N*-terminal Fmoc protected peptides with the nitroso metabolite. This procedure was followed by Fmoc deprotection and HPLC purification. The final products were essentially free of soluble dapsone, nitroso dapsone or unmodified peptide based on HPLC analysis (Figure 2A). LC-MS/MS analysis of the final purified dapsone nitroso-modified peptides revealed doubly charged ions at m/z 585.235, corresponding to the peptides with a sulphonamide adduct (Δm=278). The peptide sequence was confirmed by the presence of a series of y and b ions. The modification site was confirmed by a series of adducted b ions, all with a mass addition of 278 amu, giving evidence of modification at cysteine residue (Figure 2B & 2C). As expected, further oxidation of the sulpfonamide adduct could result in a *N*-hydroxyl sulfonamide adduct with a mass addition of 294 amu. This adduct was confirmed by the MS/MS spectrum of Pep3 AQDAEACAL as shown in Figure 2D. To explore how the nitroso dapsone-modification affects the binding affinities of the peptides two approaches were adopted. First, the cysteine residue of all three peptides were replaced with bulky aromatic amino acids (phenylalanine [F] and tryptophan [W]) to mimic the nitroso dapsone modification in the cysteine containing peptide and B*13:01 binding affinity was assessed using NetMHCpan BA 4.1. It was not possible to assess drug peptide modifications directly. Second, *in silico* models of C and W containing peptides binding to HLA-B*13:01 were generated and compared to the predicted binding of the equivalent nitroso dapsone-modified peptide. Interestingly, when the cysteine residues of the designer peptides were replaced with the bulky aromatic amino acids, the binding of these peptides to HLA-B*13:01 was stronger than the cysteine containing peptides (Figure 3A), suggesting that nitroso dapsone-modified peptides would be good binders to HLA-B*13:01. Molecular docking of AQDCEAAAL, AQDWEAAAL, and AQDC(DDS)EAAAL to a homology model of HLA-B*13:01 demonstrated that all these peptides could bind to HLA-B*13:01 similarly within the binding groove (Figure 3B). Of particular interest, the predicted conformation of dapsone modified peptide AQDC(DDS)EAAAL is similar to the peptide AQDWEAAAL (Figures 3C and D), with the bulky aromatic groups pointing out the binding groove to ensue favourable interaction of P2 and P9 anchor residues with HLA-B*13:01.

### Generation and phenotypic assessment of nitroso dapsone-modified Pep1 and Pep3 T-cell clones

**Figure 4:**
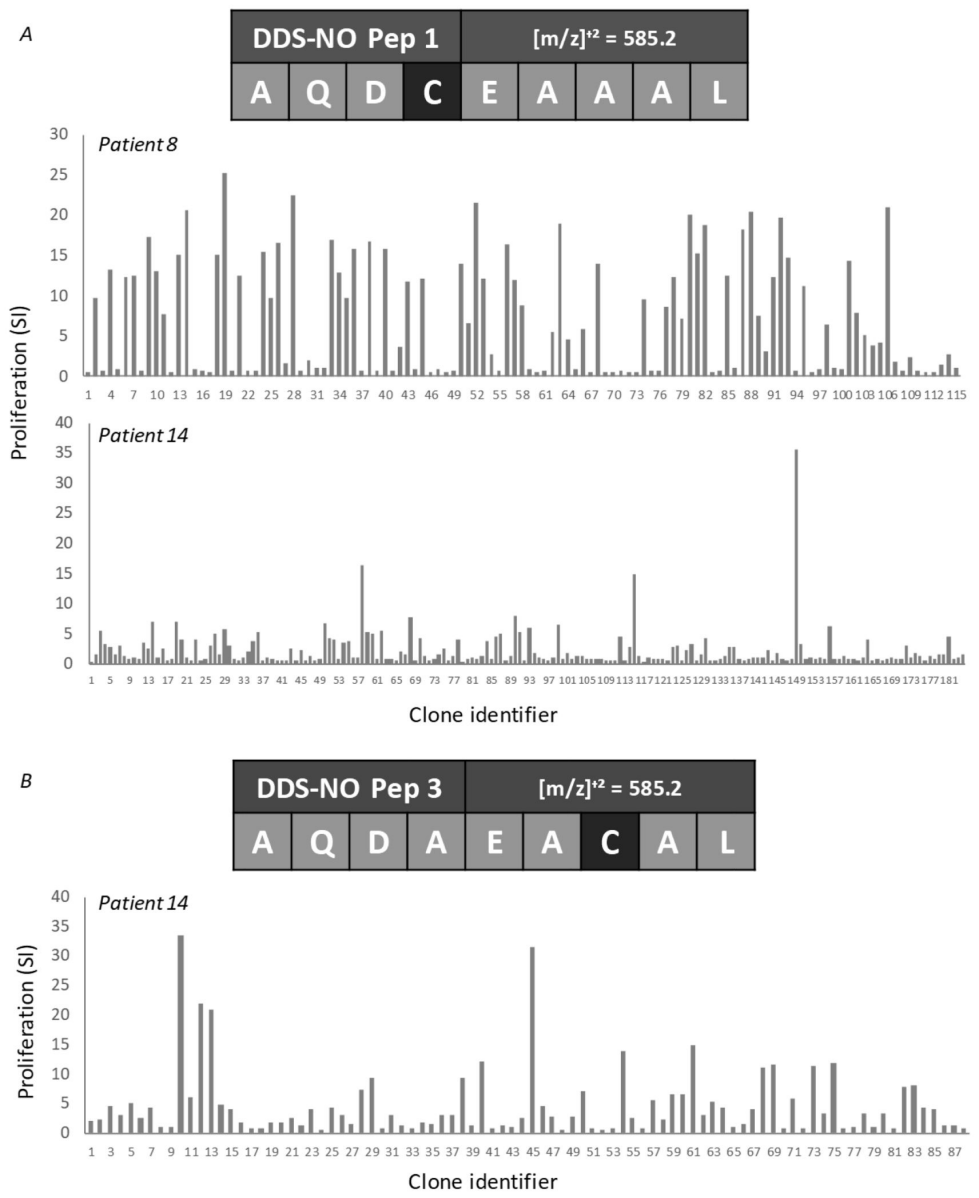
Generation of nitroso dapsone-modified Pep1- and Pep3-responsive T-cell clones from HLA-B*13:01+ dapsone hypersensitive patients. T-cell clones were generated from patient 8 or patient 14 PBMC cultures by serial dilution and repetitive mitogen stimulation. Expanded clones (5 × 10^4^ cells/well; 200 μl) were incubated in duplicate with irradiated autologous antigen presenting cells (EBV-transformed B-cells; 1 × 10^4^ cells/well) and nitroso dapsone-modified (A) Pep1 or (B) Pep3 in duplicate for 48 hrs. Proliferation was measured using [3H]-thymidine (0.5 μCi/well). Co-efficient of variation consistently less than 20%. T-cell clones with a stimulation index (SI) of > 2 were expanded for further experimental studies.

Initial testing of almost 400 T-cell clones derived from nitroso dapsone-modified Pep1 or Pep3-treated PBMC involved culture of T-cells with antigen presenting cells and the peptides or medium (as a negative control) in duplicate culture and comparison of proliferation. Almost 50% of the clones generated displayed reactivity against either nitroso dapsone-modified Pep1 (n=124) or Pep3 (n=48) and the strength of the proliferative response varied from a stimulation index (proliferation in test incubations with antigen / proliferation in control incubations with medium) of 2 to above 30 (Figure 4). These T-cell clones were expanded and analysed for CD phenotype. Clones expressing the CD8+ receptor, with no CD4 expression were used in the experiments described below.

### Functional characteristics of nitroso dapsone-modified Pep1- and Pep3-responsive CD8+ T-cell clones

**Figure 5:**
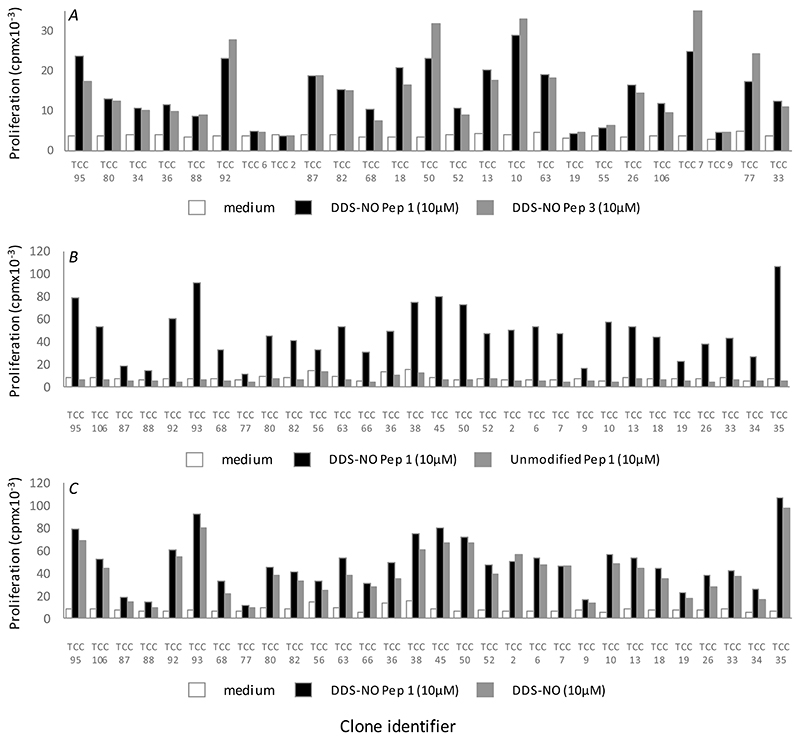
Cross-reactivity of nitroso dapsone-modified peptide-responsive CD8+ T-cell clones. A panel of up to 30 nitroso dapsone-modified peptide-responsive CD8+ clones were used to explore cross-reactivity with unmodified peptide, soluble nitroso dapsone and modified peptides with cysteine located in different positions in the peptide sequence. Clones (5 × 10^4^ cells/well; 200 μl) were incubated with irradiated autologous antigen presenting cells (EBV-transformed B-cells; 1 × 10^4^ cells/well) and (A) nitroso dapsone-modified Pep1 or nitroso dapsone-modified Pep3; (B) nitroso dapsone-modified Pep1 or unmodified Pep1; or (C) nitroso dapsone-modified Pep1 or soluble nitroso dapsone, in triplicate for 48 hrs. Proliferation was measured using [3H]-thymidine (0.5 mCi/well). Results are expressed as the mean of triplicate CPM values. Co-efficient of variation consistently less than 20%.

A panel of up to 30 T-cell clones from both patients were utilized to assess cross-reactivity. All clones were stimulated to proliferate with nitroso dapsone-modified Pep1 or Pep3 with no discernible difference between the strength of the induced response observed (Figure 5A); however, proliferative responses were not detected when the clones were cultured with unmodified peptides (Figure 5B; shows Pep1 data). All clones were also activated with soluble nitroso dapsone, which forms adducts with protein in the cell culture assay (Figure 7B), and the strength of the maximal response induced was similar with nitroso dapsone and nitroso dapsone-modified peptides (Figure 5C).

**Figure 6:**
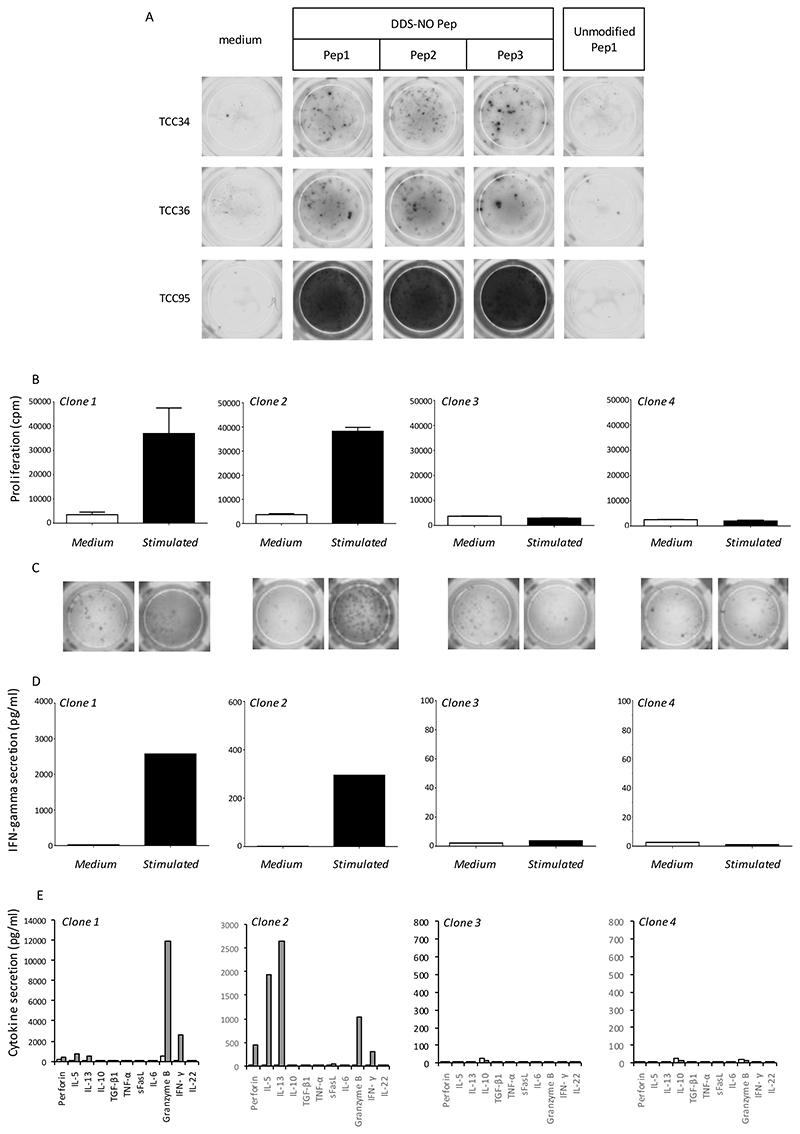
Activation of CD8+ T-cell clones with nitroso dapsone-modified Pep1, Pep2 and Pep3. (A) Nitroso dapsone-modified peptide-responsive CD8+ clones (5 × 10^4^ cells/well; 200 μl) were incubated with irradiated autologous antigen presenting cells (EBV-transformed B-cells; 1 × 10^4^ cells/well) and nitroso dapsone-modified Pep1, Pep2 or Pep3 for 48 hrs. IFN-γ release was measured by ELIspot. To demonstrate that the T-cell clones secreted a profound T-cell response, secreted cytokines were measured from 2 clones that were stimulated to proliferate and 2 that did not (B) using ELIspot (C) and a cytokine bead array (D and E). The clones that proliferated and secreted IFN-γ measured by ELIspot, were found to secrete IFN-γ using the bead array (D). These clones also secreted IL-5, Il-13, perforin and granzyme B (E).

Three clones were used in an ELIspot assay to study IFN-γ with Pep2. Clones secreted IFN-γ in the presence of all 3 nitroso dapsone-modified peptides (Pep1, Pep2 and Pep3; Figure 6A; the number of experiments with Pep2 was limited as the peptide was synthesized in small quantities). To confirm levels of cytokine produced, the supernatant from 4 clones (2 drug-specific, 2 not drug specific) was analysed using a cytokine bead array. Clones that were stimulated to proliferate also secreted IFN-γ, IL-5, Il-13, perforin and granzyme B (figure 6B-E).

Given the complete cross-reactivity profile of the clones with different peptides, well growing clones from either patient were selected and tested with Pep1 or Pep3 for the HLA restriction studies detailed below.

### Nitroso dapsone-modified peptide-responsive CD8+ T-cell clones are not activated with residual dapsone or nitroso dapsone

**Figure 7:**
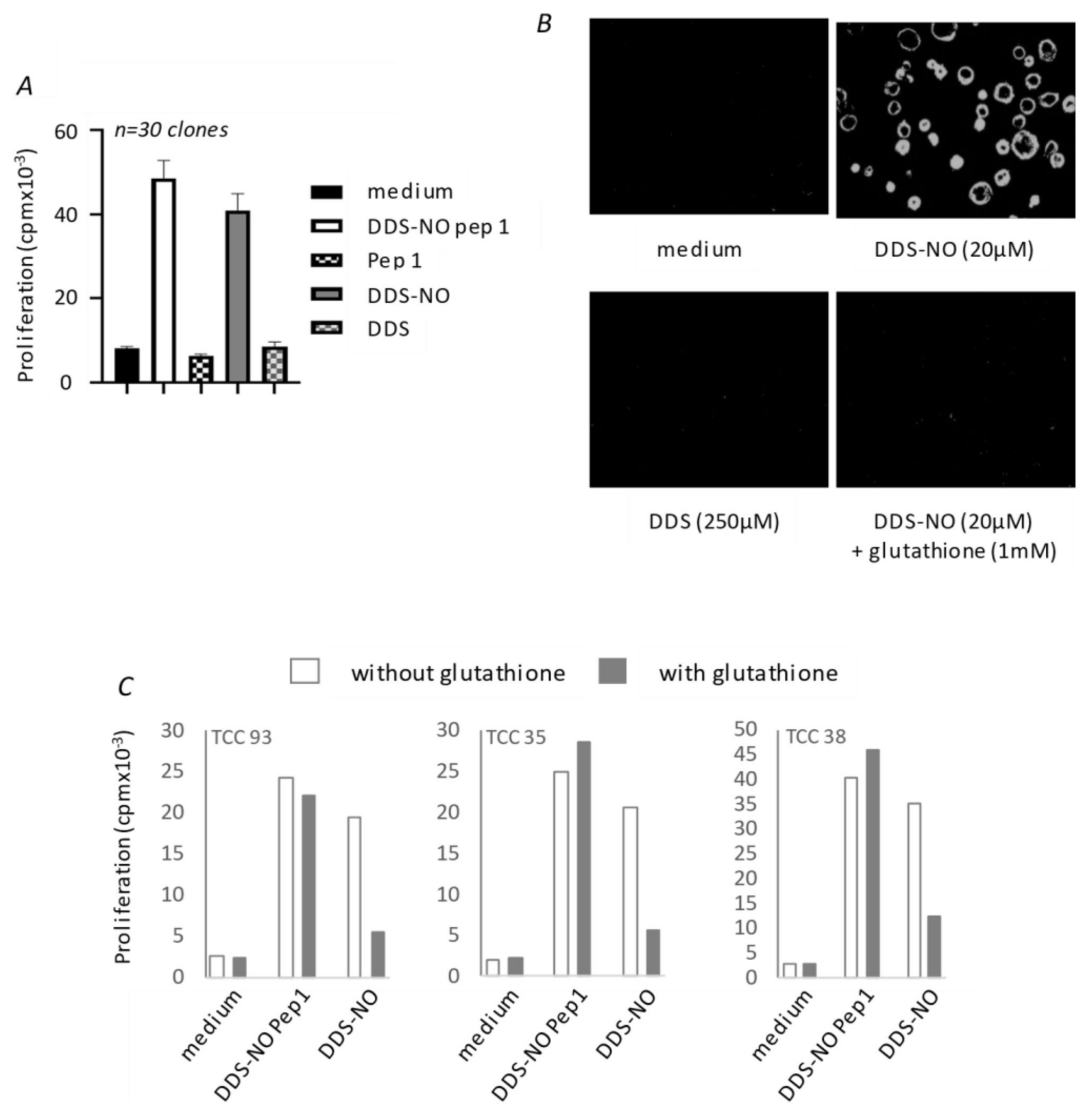
Activation of nitroso dapsone-modified peptide-responsive CD8+ T-cell clones was not due to the presence of residual dapsone or nitroso dapsone or degradation of the peptides and liberation of free dapsone. (A) To explore whether dapsone activates nitroso dapsone-modified peptide-responsive CD8+ T-cell clones, a panel of 30 clones (5 × 10^4^ cells/well; 200 μl) were incubated with irradiated autologous antigen presenting cells (EBV-transformed B-cells; 1 × 10^4^ cells/well) and dapsone at a previously defined optimal concentration (125 μM; (13)) for 48 hrs. Nitroso dapsone-modified Pep1, soluble nitroso dapsone and unmodified Pep1 were added as controls. Proliferation was measured through addition of [3H]-thymidine (0.5 μCi/well) for the final 16 hrs of the culture period. (B) Dapsone and nitroso dapsone (in the presence and absence of glutathione) were cultured with EBV-transformed B-cells and formation of covalently-modified adducts was visualised using an anti-dapsone antibody. (C) Clones (5 × 10^4^ cells/well; 200 μl) were cultured with irradiated autologous antigen presenting cells (EBV-transformed B-cells; 1 × 10^4^ cells/well) and nitroso dapsone modified Pep1 or soluble dapsone in the presence of absence of glutathione (1 mM). As shown in (B) glutathione blocks the protein reactivity of nitroso dapsone. Proliferation was measured through addition of [3H]-thymidine (0.5 μCi/well) for the final 16 hrs of the culture period.

To confirm that T-cell activation with the nitroso dapsone-modified peptides was not due to residual dapsone, clones were cultured with an optimal concentration of the parent drug. Clones were stimulated to proliferate with nitroso dapsone-modified peptide and soluble nitroso dapsone; however, proliferative responses were not detected with dapsone itself (Figure 7A). Glutathione was used to differentiate between nitroso dapsone-modified peptide and soluble nitroso dapsone T-cell proliferative responses. The addition of glutathione to cell culture medium inhibits the covalent binding of nitroso dapsone to EBV-transformed B-cells (Figure 7B) and the activation of clones with soluble nitroso dapsone. In contrast, glutathione did not alter the activation of clones with nitroso dapsone-modified peptides, where the nitroso moiety is already bound covalently to the cysteine residue in the peptide sequence (Figure 7C).

### Activation of CD8+ T-cell clones with nitroso dapsone-modified peptides is HLA-B*13:01-restricted

**Figure 8:**
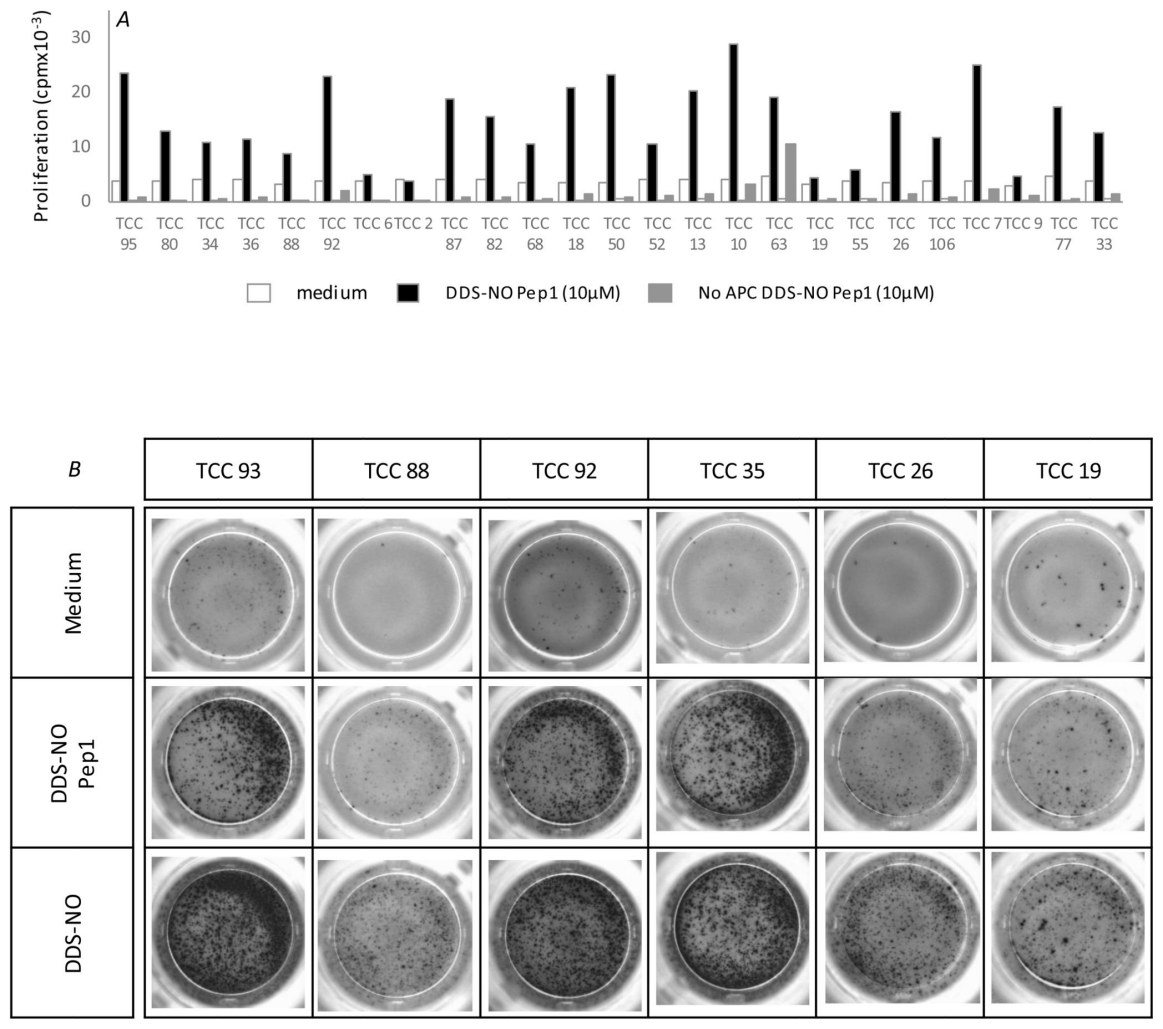
Assessment of the importance of antigen presenting cells in the activation of nitroso dapsone-modified peptide-responsive CD8+ T-cell clones. (A) Clones (5 × 10^4^ cells/well; 200 μl) were cultured with nitroso dapsone-modified Pep1 in the presence and absence of irradiated autologous antigen presenting (EBV-transformed B-cells; 1 × 10^4^ cells/well) in triplicate cultures for 48 h. Proliferation was measured through addition of [3H]-thymidine (0.5 μCi/well) for the final 16 hrs of the culture period. (B) Clones (5 × 10^4^ cells/well; 200 μl) were incubated with C1R-B*13:01 transduced antigen presenting cells (1 × 10^4^ cells/well) and either nitroso dapsone-modified Pep1 or nitroso dapsone for 48 hrs. IFN-γ release was visualized by ELIspot.

**Figure 9:**
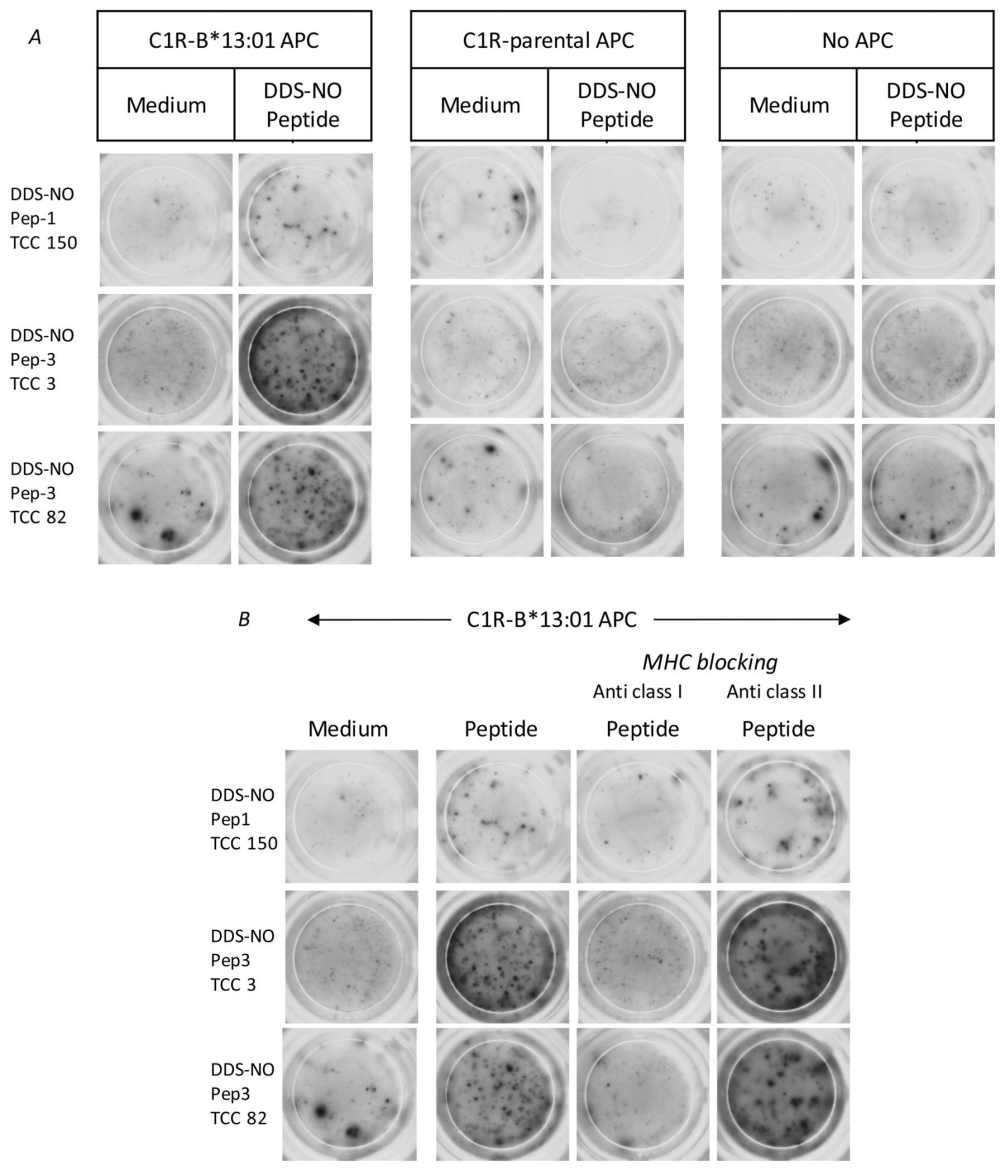
Assessment of HLA-B-13:01 restriction of the nitroso dapsone-modified peptide responsive CD8+ T-cell clones. (A) Nitroso dapsone-modified peptide-responsive clones were cultured nitroso dapsone-modified Pep1 or Pep3 and either C1R-B*13:01 or C1R-parental antigen presenting cells. Clones cultured with nitroso dapsone-modified Pep1 or Pep3 in the absence of antigen presenting cells served as a negative control. (B) Nitroso dapsone-modified peptide-responsive CD8+ clones (5 × 10^4^ cells/well; 200 μl) were incubated with C1R-B*13:01 antigen presenting cells (1 × 10^4^ cells/well) and nitroso dapsone-modified Pep1 or Pep3 in the presence or absence of anti-HLA class I and II blocking antibodies for 48 hrs. IFN-γ release was measured by ELIspot.

In *in vitro* culture conditions, soluble nitroso dapsone interacts with multiple HLA proteins to activate CD4+ and CD8+ clones from hypersensitive patients (14). The optimized culture conditions with extensive covalent modification of cellular protein potentially overrides the exquisite HLA restriction observed in patients. Thus, a stepwise approach was used to explore the restriction of the nitroso dapsone-modified peptide-specific T-cell response. First, with the exception of a small number of self-presenting clones, T-cells were not stimulated to proliferate with nitroso dapsone-modified peptides when antigen presenting cells (EBV-transformed B-cells) were excluded from the assays (Figure 8A). Second, C1R-B13:01 antigen presenting cells, expressing HLA-B*13:01, but not the other HLA class I alleles expressed by the patients were used as antigen presenting cells in the place of autologous EBV-transformed B-cells. Clones were activated and secreted IFN-γ when cultured with either nitroso dapsone-modified peptides or soluble nitroso dapsone and C1R-B*13:01 cells (Figure 8B). Third, IFN-γ secretion above control levels was not detected when the experiment was repeated with nitroso dapsone-modified Pep1 or Pep3 and C1R-parental cells (Figure 9A). Finally, pre-treatment of C1R-B*13:01 antigen presenting cells with an anti-HLA class I blocking antibody inhibited peptide-induced IFN-γ secretion, whereas an anti-HLA class II blocking antibody had no effect (Figure 9B).

## Discussion

Delayed-typed drug hypersensitivity reactions are a serious form of adverse event and represent a challenge to healthcare professionals attempting to delineate patient susceptibility. Drug-responsive T-cells are believed to be the primary effector cells involved in the iatrogenic disease, with drug (metabolite) protein or peptide binding believed to be the molecular initiating event. This interaction may involve the drug molecule binding covalently to cellular or serum proteins, as is the case for β-lactam antibiotics such as flucloxacillin (18–20). The resultant adducts are thought to be processed by antigen presenting cells into peptide fragments that associate with HLA proteins for presentation to T-cells. At the opposite end of the spectrum, drugs such as carbamazepine form labile binding interactions with HLA proteins or peptides within the HLA antigen binding cleft to stimulate a similar effector T-cell response (21–23). It should be noted that although the pathways that lead to drug display by HLA proteins differ, the chemical composition and 3D arrangement of molecules at the immunological synapse may be similar with the only difference being the nature of the drug peptide binding interaction.

Different forms of drug hypersensitivity reaction are strongly associated with expression of specific HLA class I alleles (24–26). This suggests that a derivative of the drug may interact with exquisite selectivity with the protein encoded by the HLA allele to activate the T-cells that instigate the hypersensitivity reaction. Indeed, for the archetypal association between HLA-B*57:01 and abacavir hypersensitivity (27–29), the drug adheres deep within the peptide binding cleft of HLA-B*57:01, altering the structure and the peptides that are displayed by the HLA protein to CD8+ T-cells (30–32). A similar binding interaction is not observed with closely-related HLA proteins. Regrettably, the picture is not so clear for other forms of HLA class I allele-restricted forms of drug hypersensitivity reaction. Even with exemplars such as carbamazepine (HLA-B15:02 (33), HLA-A*31:01 (34)) and flucloxacillin (HLA-B*57:01 (35)) the parent drug, drug metabolites and/or peptide adducts interact with multiple HLA class I and class II proteins to stimulate CD4+ and CD8+ T-cells in hypersensitive patients (36–41). In recent years we have focused on dapsone hypersensitivity to further define pathways of T-cell activation as (i) the metabolism and protein reactivity of dapsone is well defined (8, 9, 11), and (ii) dapsone hypersensitivity is strongly associated with HLA-B*13:01 expression (4). Dapsone and nitroso dapsone activate polyclonal CD4+ and CD8+ T-cells via different pathways, pharmacological HLA binding and hapten binding, respectively (13, 14). A number of T-cells display dapsone and nitroso dapsone cross-reactivity; however, others are highly selective in that they are stimulated with one molecule and not the other. Thus, exposure of susceptible patients to parent drug and metabolite results in the development of divergent T-cell responses that act together produce the adverse event. HLA-B*13:01-restricted dapsone- and nitroso dapsone-responsive CD8+ T-cells are detectable in assays utilizing antigen presenting from donors expressing matching HLA-B alleles. Nitroso dapsone activates T-cells via two pathways. First, through direct covalent modification of peptides embedded within MHC expressed on the surface of antigen presenting cells; and, second, through formation of protein adducts that undergo antigen processing to generate peptides that associate with MHC before transport to the cell surface for presentation to T-cells. The same clone may be activated by both pathways with the surface peptide adduct presumably mimicking the adduct formed naturally through protein processing. No information is available regarding the nature of protein adducts that activate T-cells (including whether they are formed intra or extracellularly), the different uptake pathways involved in internalising adducts and the enzymes involved in breakdown of the adducts. For this reason, we have designed and synthesized nitroso dapsone-modified HLA binding peptides to study the HLA-B*13:01-restricted T-cell response. Three nitroso dapsone-modified peptides were synthesized in high purity. Each peptide contained 2 HLA-B*13:01 anchoring motifs, an alanine backbone (previous studies show that non-anchoring amino acids for the most part do not define the specificity of the T-cell response (42, 43)) and a nucleophilic cysteine residue for modification by nitroso dapsone. An Fmoc protecting group, which was removed before purification, was used to prevent nitroso dapsone *N*-terminal binding. Reasonable yields of nitroso dapsone-modified Pep1 and Pep3 were obtained after HPLC purification; Pep2 was generated in a lower quantity and as such only used in limited T-cell cross-reactivity studies. Pep1 contains a cysteine residue in the 4 position, which is an important trinitrophenol hapten binding site for the generation of immunodominant CD8+ peptide epitopes (44). In contrast, trinitrophenol modification at more distal positions (e.g., position 7 in Pep3) generates qualitatively different determinants that tend to activate a lower frequency of T-cells (44). Modelling revealed that the predicted conformation of dapsone-modified peptides contained the bulky drug aromatic groups pointing out the binding groove to ensue favourable interaction of P2 and P9 anchor residues with HLA-B*13:01.

Two lymphocyte transformation test positive (dapsone and nitroso dapsone) hypersensitive patients (described in (14)) expressing HLA-B*13:01 were used to generate nitroso dapsone-modified peptide responsive T-cell clones. Almost 50% of clones generated from 14-day nitroso dapsone-modified peptide PBMC cultures were stimulated to proliferate in the presence of either modified Pep1 or Pep3. Clones expressed a CD8+ phenotype and were stimulated with the modified peptides in a dose-dependent manner. Clones displayed 100% cross-reactivity between positional derivatives and with soluble nitroso dapsone. Nitroso dapsone extensively modified the surface of antigen presenting cells, which likely includes binding to cysteine-containing peptides already displayed by HLA class I on the cell surface; hence, the observed cross-reactivity was expected. In contrast to our cross-reactivity data with nitroso dapsone-modified peptides, cross-reactivity between HLA class II binding β-lactam-modified peptide positional derivatives was not observed (16, 45). However, a system utilized by Honda et al (46), where CD8+ T-cell receptor α and β chains were alternately fixed prior to assessment of trinitrophenol-modified peptide positional derivative T-cell responses explains how a single T-cell receptor is capable of recognizing and responding to hapten structures in different positions. They demonstrated that hapten addition to HLA class I binding peptides (at positions 4 and 6) can cause substantial adjustments to the CD8+ T-cell receptor structure Specifically, the β chain could adjust to interact with the hapten structure irrespective of whether it was at position 4 or 6.

The possibility that the T-cell activation with nitroso dapsone-modified peptides may be due to residual soluble nitroso dapsone or degradation of the adduct in culture and liberation of dapsone was excluded through (i) demonstrating that the clones were not activated with the parent compound, and (ii) neutralizing soluble nitroso dapsone-specific, but not nitroso dapsone-modified peptide-specific, T-cell responses with glutathione. Glutathione contains a reactive cysteine group and when in excess binds to nitroso dapsone, preventing formation of protein adducts.

To explore the importance of HLA proteins in T-cell activation, antigen presenting cells were firstly excluded from T-cell proliferation assays. The vast majority of clones were not activated with the nitroso dapsone-modified peptides in the absence of antigen presenting cells. Next, the HLA-A, B negative mutant C1R cell line was transduced with HLA-B*13:01 and used as antigen presenting cells in the place of autologous EBV-transformed B-cells. Nitroso dapsone-modified Pep1 and Pep3, and soluble nitroso dapsone stimulated the clones to secrete IFN-γ in the presence of C1R-B*13:01 cells and the response was inhibited with an HLA class I blocking antibody. Similar activation of the T-cell clones was not observed when using the C1R parental cell line as antigen presenting cells.

Collectively, our study highlights the importance of drug metabolism, drug hapten binding and most importantly the formation of drug metabolite-modified HLA-B*13:01 binding peptides in the activation of CD8+ T-cells from dapsone hypersensitive patients. The availability of T-cell stimulatory nitroso dapsone-modified HLA-B*13:01 binding peptides and C1R-B*13:01 cells offer the opportunity to study the structural elements of the drug hapten HLA peptide binding interaction.

## Key points

1. Nitroso dapsone-modified HLA binding peptides activate human CD8+ T-cells
2. T-cell activation was restricted to HLA-B*13:01.

## Abbreviations

SI: stimulation index
HLA: human leukocyte antigen
PBMC: peripheral blood mononuclear cells
cpm: counts per minute.

## References

[1] Allday EJ, Barnes J (1951). Toxic effects of diaminodiphenylsulphone in treatment of leprosy. Lancet.

[2] Liu H, Wang Z, Bao F, Wang C, Sun L, Zhang H, Yu G, Mi Z, Li J, Li L, Zhao Q (2019). Evaluation of Prospective HLA-B*13:01 Screening to Prevent Dapsone Hypersensitivity Syndrome in Patients With Leprosy. JAMA Dermatol.

[3] Wang H, Yan L, Zhang G, Chen X, Yang J, Li M, Shen MJ, Yu M, Wang B (2013). Association between HLA-B*1301 and dapsone-induced hypersensitivity reactions among leprosy patients in China. J Invest Dermatol.

[4] Zhang FR, Liu H, Irwanto A, Fu XA, Li Y, Yu GQ, Yu YX, Chen MF, Low HQ, Li JH, Bao FF (2013). HLA-B*13:01 and the dapsone hypersensitivity syndrome. N Engl J Med.

[5] Satapornpong P, Pratoomwun J, Rerknimitr P, Klaewsongkram J, Nakkam N, Rungrotmongkol T, Konyoung P, Saksit N, Mahakkanukrauh A, Amornpinyo W, Khunarkornsiri U (2021). HLA-B*13 :01 is a predictive marker of dapsone-induced severe cutaneous adverse reactions in Thai patients. Front Immunol.

[6] Park HJ, Park JW, Kim SH, Choi SY, Kim HK, Jung CG, Yang MS, Kang DY, Cho MK, Kwon HS, Kang HR (2020). The HLA-B*13:01 and the dapsone hypersensitivity syndrome in Korean and Asian populations: genotype- and meta-analyses. Expert Opin Drug Saf.

[7] Gill HJ, Tingle MD, Park BK (1995). N-hydroxylation of dapsone by multiple enzymes of cytochrome P450: implications for inhibition of haemotoxicity. Br J Clin Pharmacol.

[8] Reilly TP, Lash LH, Doll MA, Hein DW, Woster PM, Svensson CK (2000). A role for bioactivation and covalent binding within epidermal keratinocytes in sulfonamide-induced cutaneous drug reactions. J Invest Dermatol.

[9] Roychowdhury S, Vyas PM, Reilly TP, Gaspari AA, Svensson CK (2005). Characterization of the formation and localization of sulfamethoxazole and dapsone-associated drug-protein adducts in human epidermal keratinocytes. J Pharmacol Exp Ther.

[10] Vyas PM, Roychowdhury S, Koukouritaki SB, Hines RN, Krueger SK, Williams DE, Nauseef WM, Svensson CK (2006). Enzyme-mediated protein haptenation of dapsone and sulfamethoxazole in human keratinocytes - 2. Expression and role of flavin-containing monooxygeanses and peroxidases. J Pharmacol Exp Ther.

[11] Alzahrani A, Ogese M, Meng X, Waddington JC, Tailor A, Farrell J, Maggs JL, Betts C, Park BK, Naisbitt DJ (2017). Dapsone and Nitroso Dapsone Activation of Naive T-Cells from Healthy Donors. Chem Res Toxicol.

[12] Chen WT, Wang CW, Lu CW, Chen CB, Lee HE, Hung SI, Choon SE, Yang CH, Liu MT, Chen TJ, Fan WL (2018). The function of HLA-B*13:01 involved in the pathomechanism of dapsone-induced severe cutaneous adverse reactions. J Invest Dermatol.

[13] Zhao Q, Alhilali K, Alzahrani A, Almutairi M, Amjad J, Liu H, Sun Y, Sun L, Zhang H, Meng X, Gibson A (2019). Dapsone- and nitroso dapsone-specific activation of T cells from hypersensitive patients expressing the risk allele HLA-B*13:01. Allergy.

[14] Zhao Q, Almutairi M, Tailor A, Lister A, Harper N, Line J, Meng X, Pratoomwun J, Jaruthamsophon K, Sukasem C, Sun Y (2021). HLA Class-II restricted CD8(+) T Cells contribute to the promiscuous immune response in dapsone-hypersensitive patients. J Invest Dermatol.

[15] Reynisson B, Alvarez B, Paul S, Peters B, Nielsen M (2020). NetMHCpan-4.1 and NetMHCIIpan-4.0: improved predictions of MHC antigen presentation by concurrent motif deconvolution and integration of MS MHC eluted ligand data. Nucleic Acids Res.

[16] Tailor A, Meng X, Adair K, Farrell J, Waddington JC, Daly A, Pirmohamed M, Dear G, Park BK, Naisbitt DJ (2020). HLA DRB1*15:01-DQB1*06:02-restricted human CD4+ T cells are selectively activated with amoxicillin-peptide adducts. Toxicol Sci.

[17] Manchanda T, Hess D, Dale L, Ferguson SG, Rieder MJ (2002). Haptenation of sulfonamide reactive metabolites to cellular proteins. Mol Pharmacol.

[18] Puig M, Ananthula S, Venna R, Kumar Polumuri S, Mattson E, Walker LM, Cardone M, Takahashi M, Su S, Boyd LF, Natarajan K (2020). Alterations in the HLA-B*57:01 immunopeptidome by flucloxacillin and immunogenicity of drug-haptenated peptides. Front Immunol.

[19] Monshi MM, Faulkner L, Gibson A, Jenkins RE, Farrell J, Earnshaw CJ, Alfirevic A, Cederbrant K, Daly AK, French N, Pirmohamed M (2013). Human leukocyte antigen (HLA)-B*57:01-restricted activation of drug-specific T cells provides the immunological basis for flucloxacillin-induced liver injury. Hepatology.

[20] Waddington JC, Meng X, Illing PT, Tailor A, Adair K, Whitaker P, Hamlett J, Jenkins RE, Farrell J, Berry N, Purcell AW (2020). Identification of flucloxacillin-haptenated HLA-B*57:01 ligands: evidence of antigen processing and presentation. Toxicol Sci.

[21] Wei CY, Chung WH, Huang HW, Chen YT, Hung SI (2012). Direct interaction between HLA-B and carbamazepine activates T cells in patients with Stevens-Johnson syndrome. J Allergy Clin Immunol.

[22] Naisbitt DJ, Britschgi M, Wong G, Farrell J, Depta JP, Chadwick DW, Pichler WJ, Pirmohamed M, Park BK, Naisbitt DJ (2003). Hypersensitivity reactions to carbamazepine: characterization of the specificity, phenotype, and cytokine profile of drug-specific T cell clones. Mol Pharmacol.

[23] Wu Y, Sanderson JP, Farrell J, Drummond NS, Hanson A, Bowkett E, Berry N, Stachulski AV, Clarke SE, Pichler WJ, Pirmohamed M (2006). Activation of T cells by carbamazepine and carbamazepine metabolites. J Allergy Clin Immun.

[24] Deshpande P, Hertzman RJ, Palubinsky AM, Giles JB, Karnes JH, Gibson A, Phillips EJ (2021). Immunopharmacogenomics: Mechanisms of HLA-associated drug reactions. Clin Pharmacol Ther.

[25] Jaruthamsophon K, Thomson PJ, Sukasem C, Naisbitt DJ, Pirmohamed M (2021). HLA allele-restricted immune-mediated adverse drug reactions: framework for genetic prediction. Annu Rev Pharmacol Toxicol.

[26] Nicoletti P, Aithal GP, Bjornsson ES, Andrade RJ, Sawle A, Arrese M, Barnhart HX, Bondon-Guitton E, Hayashi PH, Bessone F, Carvajal A (2017). Association of liver injury from specific drugs, or groups of drugs, with polymorphisms in HLA and other genes in a genome-wide association study. Gastroenterology.

[27] Mallal S, Phillips E, Carosi G, Molina JM, Workman C, Tomazic J, Jägel-Guedes E, Rugina S, Kozyrev O, Cid JF, Hay P (2008). HLA-B*5701 screening for hypersensitivity to abacavir. N Engl J Med.

[28] Mallal S, Nolan D, Witt C, Masel G, Martin AM, Moore C, Sayer D, Castley A, Mamotte C, Maxwell D, James I (2002). Association between presence of HLA-B*5701, HLA-DR7, and HLA-DQ3 and hypersensitivity to HIV-1 reverse-transcriptase inhibitor abacavir. Lancet.

[29] Hetherington S, Hughes AR, Mosteller M, Shortino D, Baker KL, Spreen W, Lai E, Davies K, Handley A, Dow DJ, Fling ME (2002). Genetic variations in HLA-B region and hypersensitivity reactions to abacavir. Lancet.

[30] Illing PT, Vivian JP, Dudek NL, Kostenko L, Chen Z, Bharadwaj M, Miles JJ, Kjer-Nielsen L, Gras S, Williamson NA, Burrows SR (2012). Immune self-reactivity triggered by drug-modified HLA-peptide repertoire. Nature.

[31] Ostrov DA, Grant BJ, Pompeu YA, Sidney J, Harndahl M, Southwood S, Oseroff C, Lu S, Jakoncic J, de Oliveira CA, Yang L (2012). Drug hypersensitivity caused by alteration of the MHC-presented self-peptide repertoire. Proc Natl Acad Sci U S A.

[32] Norcross MA, Luo S, Lu L, Boyne MT, Gomarteli M, Rennels AD, Woodcock J, Margulies DH, McMurtrey C, Vernon S, Hildebrand WH (2012). Abacavir induces loading of novel self-peptides into HLA-B*57: 01: an autoimmune model for HLA-associated drug hypersensitivity. AIDS.

[33] Chung WH, Hung SI, Hong HS, Hsih MS, Yang LC, Ho HC, Wu JY, Chen YT (2004). Medical genetics: a marker for Stevens-Johnson syndrome. Nature.

[34] McCormack M, Alfirevic A, Bourgeois S, Farrell JJ, Kasperaviciute D, Carrington M, Sills GJ, Marson T, Jia X, de Bakker PI, Chinthapalli K (2011). HLA-A*3101 and carbamazepine-induced hypersensitivity reactions in Europeans. N Engl J Med.

[35] Daly AK, Donaldson PT, Bhatnagar P, Shen Y, Pe’er I, Floratos A, Daly MJ, Goldstein DB, John S, Nelson MR, Graham J (2009). HLA-B*5701 genotype is a major determinant of drug-induced liver injury due to flucloxacillin. Nat Genet.

[36] Monshi MM, Faulkner L, Gibson A, Jenkins RE, Farrell J, Earnshaw CJ, Alfirevic A, Cederbrant K, Daly AK, French N, Pirmohamed M (2013). Human leukocyte antigen (HLA)-B*57:01-restricted activation of drug-specific T cells provides the immunological basis for flucloxacillin-induced liver injury. Hepatology.

[37] Yaseen FS, Saide K, Kim SH, Monshi M, Tailor A, Wood S, Meng X, Jenkins R, Faulkner L, Daly AK, Pirmohamed M (2015). Promiscuous T-cell responses to drugs and drug-haptens. J Allergy Clin Immunol.

[38] Wuillemin N, Adam J, Fontana S, Krahenbuhl S, Pichler WJ, Yerly D (2013). HLA haplotype determines hapten or p-i T cell reactivity to flucloxacillin. J Immunol.

[39] Lichtenfels M, Farrell J, Ogese MO, Bell CC, Eckle S, McCluskey J, Park BK, Alfirevic A, Naisbitt DJ, Pirmohamed M (2014). HLA restriction of carbamazepine-specific T-Cell clones from an HLA-A*31:01-positive hypersensitive patient. Chem Res Toxicol.

[40] Ko TM, Chung WH, Wei CY, Shih HY, Chen JK, Lin CH, Chen YT, Hung SI (2011). Shared and restricted T-cell receptor use is crucial for carbamazepine-induced Stevens-Johnson syndrome. J Allergy Clin Immunol.

[41] Wu Y, Farrell J, Pirmohamed M, Park BK, Naisbitt DJ (2007). Generation and characterization of antigen-specific CD4+, CD8+, and CD4+CD8+ T-cell clones from patients with carbamazepine hypersensitivity. J Allergy Clin Immunol.

[42] Ortmann B, Martin S, von Bonin A, Schiltz E, Hoschutzky H, Weltzien HU (1992). Synthetic peptides anchor T cell-specific TNP epitopes to MHC antigens. J Immunol.

[43] Martin S, Ortmann B, Pflugfelder U, Birsner U, Weltzien HU (1992). Role of hapten-anchoring peptides in defining hapten-epitopes for MHC-restricted cytotoxic T cells. Cross-reactive TNP-determinants on different peptides. J Immunol.

[44] Martin S, von Bonin A, Fessler C, Pflugfelder U, Weltzien HU (1993). Structural complexity of antigenic derminants for class I MHC-restricted, hapten-specific T cells. J Immunol.

[45] Padovan E, Bauer T, Tongio MM, Kalbacher H, Weltzien HU (1997). Penicilloyl peptides are recognized as T cell antigenic determinants in penicillin allergy. Eur J Immunol.

[46] Honda S, Zhang W, Kalergis AM, DiLorenzo TP, Wang F, Nathenson SG (2001). Hapten addition to an MHC class I-binding peptide causes substantial adjustments of the TCR structure of the responding CD8(+) T cells. J Immunol.

